# Group versus Individual Care in Patients with Long-Standing Type 1 and Type 2 Diabetes: A One-Year Prospective Noninferiority Study in a Tertiary Diabetes Clinic

**DOI:** 10.1155/2018/1807246

**Published:** 2018-05-28

**Authors:** Joelle Singer, Sigal Levy, Ilan Shimon

**Affiliations:** ^1^Institute of Endocrinology, Diabetes and Metabolism, Beilinson Hospital, Rabin Medical Center, Jabotinski Road 39, 49100 Petah Tikva, Israel; ^2^Sackler School of Medicine, Tel Aviv University, Chaim Lebanon 30, 6997801 Tel Aviv, Israel; ^3^Statistical Education Unit, The Academic College of Tel Aviv-Yaffo, Khever ha-Le'umim St 10, Tel Aviv-Yafo, Israel

## Abstract

**Aims:**

To explore the feasibility and noninferiority of group care in a diabetes outpatient clinic in comparison with individual care.

**Methods:**

In this prospective, randomized, nonblinded, one center (university hospital) trial, 60 patients (28 with type 1 and 32 with type 2 diabetes) with a mean duration of diabetes of 22.5 ± 11.7 years were randomly assigned to group (6 patients per group) or individual care for one year. The primary endpoints were the change in HbA1c and visits to outpatient clinics. The secondary endpoints were changes in body mass index, blood pressure levels, waist circumference, non-HDL cholesterol, diabetes-related and well-being index questionnaires, and the number of hospitalizations.

**Results:**

Group care was not inferior to individual care for any of the above parameters except for the number of visits to a primary care physician.

**Conclusion:**

Group care is feasible in a diabetes clinic and is as effective as individual care. Implementation of group care may facilitate access to specialized care to a larger population of patients with diabetes type 1 and 2.

## 1. Introduction

Diabetes is a highly prevalent disorder worldwide. According to the World Health Organization (WHO), the number of patients with diabetes has risen from 108 million in 1980 to 422 million in 2014, mainly owing to a rise in the prevalence of type 2 diabetes [1]. The incidence of type 1 diabetes has been on the rise as well: from 8 new cases per 100,000 inhabitants in 1997 to 13.9 per 100,000 in 2011 in Israel [2]. Across the OECD, average annual growth in per capita health expenditure increased after 2010. The per capita health expenditure increase remains below the 2008 precrisis levels and is in line with average economic growth (growth domestic product (GDP)) for most OECD countries [3]. Resources in most health care systems did not expand at a rate parallel to the increase in the prevalence of diabetes, with a total number of people diagnosed with diabetes projected to rise from 171 million in 2000 to 366 million in 2030 [4] while there is a relative shortage in clinicians in most OECD countries [5]. New approaches are therefore needed to better deliver care in both the primary and specialist care system so as to meet the needs of this growing patient population. Adherence to long term therapies is not optimal and is influenced by many factors, including socioeconomic state, health system capabilities, health care teams, specific therapies, and other patient-related factors. In turn, these conditions must be taken into consideration in order to optimize the delivery of therapy [6]. Group care was mainly explored in patients with type 2 diabetes and in primary care settings [7]. Group care follow-up, of patients with diabetes in specialist outpatient clinics, was superior to usual care for metabolic control, prevention of deterioration of type 2 diabetes, quality of life, and reduction of cardiovascular risks [8–11]. Most of the clinical studies looking into group care in a specialist setting have been conducted in Italy by the ROMEO group [8]. Since cultural issues could influence the response to group treatment [12], and applicability of group care in type 1 diabetes was not studied [13], the present study was conducted to evaluate the feasibility and noninferiority of group care for patients with both type 1 and 2 diabetes attending a tertiary outpatient specialist clinic.

## 2. Materials and Methods

### 2.1. Ethical Statement

This prospective randomized controlled, nonblind, single-center study was performed according to the ethical standards of the World Medical Association's Declaration of Helsinki. The protocol was approved by the local IRB and registered at http://www.ClinicalTrials.gov (NCT03353376). Written informed consent was obtained from each patient before trial participation.

#### 2.1.1. Study Protocol

Patients were recruited according to the following inclusion criteria: diagnosis of type 1 or type 2 diabetes, age above 18 years, fluency in Hebrew, attendance for the second time, at least, at the diabetes clinic, follow-up period of at least one year by a primary care physician (PCP), and willingness to participate in the study and to give informed consent. The exclusion criteria included uncontrolled psychiatric diseases with hospitalization for psychiatric condition in the last year before recruitment, active cancer, or pregnancy.

Patients were randomized to either group care (GC) or individual care (IC) according to a computerized sequence generation for randomization. Randomization was done separately for patients with type 1 and type 2 diabetes.

At baseline and after one year, weight, height, waist circumference, and systolic and diastolic blood pressure (SBP and DBP, resp.) were measured, body mass index (BMI) was calculated, and patients were asked to complete three questionnaires, two evaluating patients' concerns about diabetes (personal models of diabetes (PMD) [14–17] and patient areas in diabetes (PAID) [18, 19]), and one evaluating well-being (World Health Organization quality of life short version (WHO-5) [20]). Validated Hebrew versions of the three questionnaires were used. PMD is an 8-question questionnaire exploring patient's perceived seriousness of disease, treatment effectiveness, and fatalistic ideation; PAID is a 20-item questionnaire related to emotional distress in diabetes; WHO-5 is a 5-item questionnaire regarding well-being and emotional functioning. Hebrew validation was made by translation to Hebrew and back to English.

The levels of HbA1c, non-HDL cholesterol, number of clinic visits, and the number of hospitalizations were extracted from the electronic file of the patient, while we used a mean of all values available for HbA1c and non-HDL cholesterol levels during the year preceding the study (year 1) and the study year (year 2).  Table 1 describes follow-up in both group and individual care.

Follow-up in the GC consisted of four two-hour group visits (six patients per group) during the year of the study. Patients with the same type of diabetes were followed in each group visit. Each of the two-hour visits was divided as follows:

30 minutes: a clinic nurse took anthropometric measures (weight, height, blood pressure, and waist circumference) and responded to the patient's questions, while a physician reviewed the patient's laboratory work-up.

50–60 minutes: all the patients met with the physician with no preestablished program. Issues that were of interest for the patients were discussed according to principles of group therapy and the empowerment model described by Funnel et al. [21, 22].

20–30 minutes: each patient received a letter to the primary care physician (PCP) with recommendations.

No phone calls were used as follow-up education sessions.

The same physician followed patients of GC and IC for the year of the study. Patients in the IC arm were followed according to the usual care used in our diabetes clinic. Usual individual care was comprised of a nurse examination, including anthropometric measurements, foot examination, download of glucometers and insulin pumps, and review of injection techniques when needed (around 10 minutes), and an endocrinologist consultation (around 25 minutes). The mean annual visit number in our outpatient clinic is 2.5 a year per patient.

Both primary and secondary outcomes were composite endpoints and were studied to prove the noninferiority of intervention. Primary outcomes included the difference between year 2 and year 1 for HbA1c values and number of planned and unplanned visits to the diabetes clinic and to the primary care clinic.

Secondary outcome included the difference between year 2 and year 1 for the scores of the 3 questionnaires, the number of hospitalizations, non-HDL cholesterol values, and anthropometric measures.

The results are expressed as a difference between year 2 and year 1. By differentiating between year 2 and year 1, the anthropometric and biochemical parameters negative results signify an improvement of these parameters.

#### 2.1.2. Statistical Analysis

We tested for differences in improvement between the control and study groups using a mixed-model repeated measures ANOVA, with time (baseline versus follow-up) as a within-subjects factor and group assignment as a between-subjects factor. The interaction term of this analysis served us to test whether the change over time differed between groups. Power analysis showed that the sample we used was suitable for detecting a large effect (Cohen's *D* = 0.8) with a probability of over 80%. Results were considered significant at *p* < 0.05. Statistical tests were conducted using IBM SPSS Statistics for Windows, Version 22.0. Armonk, NY: IBM Corp. Power analysis was done using G^∗^Power version 3.1.9.2.

## 3. Results

165 patients were screened between September 13, 2010 and January 2, 2014. Of them, 105 declined participation. The 60 patients that agreed to participate were randomized to the GC arm (*n* = 32, 16 patients with type 1 and 16 with type 2 diabetes) or to the IC arm (*n* = 28, 12 patients with type 1 and 16 with type 2 diabetes). The mean diabetes duration was 22.5 years ± 11.75 (range 3 to 54 years). All patients attended all visits and completed one year of study.  Table 2 shows patients' characteristics and change in HbA1c, weight, waist circumference, BMI, SBP, or DBP, and non-HDL cholesterol as well as visits to PCP and diabetes clinic between year 2 and year 1. No significant difference was observed between the GC and IC groups. All parameters showed a nonsignificant trend for improvement in both groups. The only significant difference was in the number of visits to the PCP, which was higher in the GC compared to the IC (difference in number of visits between year 2 and year 1 of −5.9 ± 8.4 in the IC group versus 0.8 ± 8.8 per year in the GC group (*p* < 0.02)). No significant difference was shown in the number of visits to the diabetes clinic.

Table 3 shows the difference between year 2 and year 1 for HbA1c, weight, BMI, SBP, DBP, non-HDL cholesterol, visits at the primary care clinic and diabetes clinic, hospital admission, and the questionnaire scores according to the type of diabetes (Table 3(a), patients with type 1; Table 3(b), patients with type 2) among the GC and IC groups. Perception of diabetes as well as well-being was not significantly different according to the PMD and WHO-5 questionnaires at year 2 between the two treatment arms. PAID questionnaire scores were significantly lower in the IC group compared to the GC (*p* < 0.03) arm. The number of hospital admissions was 0.3 ± 0.5 in the IC group and 0.9 ± 2.3 in the GC group but this difference was not found to be clinically significant. One patient that was hospitalized 16 times during the study year had in fact been hospitalized 12 times in the year prior to study enrollment.

## 4. Discussion

Our results show that group care is a feasible approach not inferior to the traditional one.

### 4.1. Health Care Resources Utilization

The IC group had a significant lower number of visits to the GP only in patients with type 2 diabetes. This finding requires further evaluation due to the fact that the attempt to improve deliverance of care is an optimization of healthcare resources, but the number of visits to the diabetes clinic was similar between the two groups, with similar personal and biochemical outcomes. This finding was not associated with an increase in hospitalization rate, one of the leading contributors to health expenses [23].

In our study, all of the patients attended the clinic with no change to usual care except for the fact that the visit was either one on one or in a group with no change in access to the nurses, dietitians, or psychologists working in our clinic. Appointment attendance rate was high compared to that found in a previous study conducted in a primary care setting [24]. In comparison to patients in the IC arm, in the GC arm, there existed a trend towards shorter consultation time for patient follow-up. The only change required in the organization of the diabetic clinic was administrative, namely a change in the physician's appointments to permit a slot of 2 hours for 6 patients, instead of a slot of 25 minutes per patient. Time spent with the diabetes team was 80 and 87 minutes per patient per year, in the group and individual care, respectively, while the number of annual contacts with the diabetes team was higher for patients receiving group care (4 contacts a year in GC and 2.5 in IC).

### 4.2. Personal and Biochemical Outcomes

The clinical outcome in the two groups showed nonsignificant trends for improvement in weight, blood pressure, glucose, and cholesterol levels with noninferiority of the group care. A Cochrane collaboration [25] reviewed group-based training for self-management strategies in people with type 2 diabetes mellitus and concluded that group-based training for self-management strategies in this patient population was effective in improving fasting blood glucose levels, glycated hemoglobin, and diabetes knowledge, while reducing systolic blood pressure levels, body weight, and the requirement for diabetes medications. Other meta-analyses showed the positive effects of group care in people with type 2 diabetes regarding glucose and HbA1c levels [26, 27]. A recent study [28], in a different setting of primary care and using the Trento model, showed an improvement in HbA1c levels. In each of the aforementioned studies [25–28], the patients were at an earlier stage of diabetes. PAID questionnaire scores were significantly lower in the IC group compared to the GC, with no difference in the two other questionnaires used. A very low score on the PAID questionnaire may reflect denial of illness or a low degree of concern. This significant difference was observed only in patients with type 1 diabetes.

### 4.3. Population

All patients with type 1 and most of the patients with type 2 were on injectable drugs and some on insulin pumps. Our study's population was different from that studied in the ROMEO as well as other studies: in these trials, group care was studied in patients at risk for type 2 diabetes with no hypoglycemic drugs [12]; at an earlier stage of the disease, requiring oral hypoglycemic drugs [5–8]; or patients with type 1 diabetes on 4 injections a day [29]. Group medical visits are under intense investigation with many randomized clinical trials underway, mainly in primary care [30]. In a study by Trento et al. [29], the number of visits was higher than in our study, and furthermore, after 9 sessions of group focus, the follow-up was planned according to a predetermined educational plan. In our study, we mainly altered the setup of the visits with no predetermined educational plan and discussed the issues that were of interest to the patients at each visit.

In our study, we followed up patients with type 1 diabetes, some of them on insulin pumps, a population not studied in previous studies held in a specialist clinic. This study gives proof of concept that group care is applicable to people with diabetes at an advanced stage of the disease or those with type 1 diabetes using multiple treatment modalities.

### 4.4. Type of Intervention

Generally speaking, group care may be provided by different healthcare professionals, including educators, primary care physicians, diabetes specialists, dietitians, and psychologists [8, 11, 13, 18], as in the landmark ROMEO studies. Many studies incorporated a multidisciplinary group care approach with a structured educational plan, even when using the empowerment method [11, 21, 22, 31, 32] that emphasizes open discussion of subjects that are of interest to the patients [33]. Many diabetes clinics employ multidisciplinary teams comprised of physicians, nurses, dietitians, and administrators, while psycho-social healthcare professionals or educators are not always available as health care providers. This implies that finding an available process of care while relying on the existing personnel may increase the implementation of proven and efficient new ways to care for the growing population of people with diabetes. In the present study, both IC and GC were performed by the same physician with no scheduled programs, allowing patients to decide on which issues to concentrate, while requiring only minimal administrative changes.

### 4.5. Strengths and Limitations

The strength of our study is that there was no caregiver variability. The study was conducted in a busy diabetes clinic (around 3500 visits a year) with no additional health care professionals other than the usual team working in the clinic. The population studied can be described as suffering from long-standing diabetes, almost half with type 1. The main limitations of our study were the relatively small number of patients and the relatively short follow-up period of one year. There is a need for larger studies to explore group therapy as a new paradigm in chronic disease follow-up and treatment.

## 5. Conclusion

Our study shows the positive impact of group care on glucose control and life of people with diabetes. Our study was conducted in a small sample of patients, and since 2014, several attempts have been made to use group therapy as standard care but have not been successful due to administrative reasons. There is no code to charge the HMOs for group treatment as we organized it during this research. A larger study may be needed to change this paradigm in the mind of the healthcare providers and HMOs. Despite available data, health systems are generally slow to adapt care paradigms in diabetes, or, in fact, chronic diseases in general. A frequency of four annual sessions, one that is frequently advocated as the standard follow-up for complicated patients with diabetes in a tertiary hospital in the diabetic clinic, may in fact allow for a higher number of patients with diabetes to be followed up in specialized diabetes clinics. Group care that is applicable to patients with type 1 and type 2 diabetes may allow for better access to specialized care when needed and should be considered as a viable option for diabetes care delivery in a diabetes clinic.

## Figures and Tables

**Table 1:** Patient follow-up according to study arm. mn = minutes.

Study arm	Visits per year	Duration of visit	Description of visit	Time spent per patient per year
Group care (GC)	4	120 mn	Nurse:30 mn Physician: group 60 mn// One on one: 5 mn per patient	80 mn
Individual care (IC)	2.5	35 mn	Nurse: 10 mn Physician: 25 mn	87 mn

**Table 2:** Characteristics of patients (age, duration of diabetes (years)) and change at one year in HbA1c (%), weight (kg), body mass index (kg/m^2^), waist circumference (cm), and systolic and diastolic blood pressure (mmHg) according to group assignment (individual care and group care). Number of visits to family physician and to diabetes clinic is expressed as difference between study year and preceding year.

Parameter	Individual care			Group care			*p* value
	*N*	Mean	SD	*N*	Mean	SD	
Age	28	60.0±	10.6	32	53.2±	15.3	0.53
Duration of diabetes	28	25.0±	13.2	29	20.2±	10.0	0.13
HbA1c start	26	8.2	1.1	33	8.3	1.2	0.70
HgA1c end	24	8.2	1.1	32	8.2	1.2	0.90
Weight start	26	83.8	11.8	31	79.5	14.7	0.23
Weight end	25	81.8	10.6	32	78.6	14.2	0.36
BMI start	26	29.8	4.8	31	28.7	4.2	0.34
BMI end	26	28.1	7.1	33	27.2	6.2	0.63
Waist start	13	110.5	11.9	16	102.1	14.4	0.10
Waist end	6	107.8	9.4	26	102.0	105	0.22
SBP start	24	124	18	30	128	17	0.46
SBP end	21	124	18	32	126	20	0.68
DBP start	24	73	11	30	74	8	0.79
DBP end	21	73	9	32	71	8	0.44
PCP visits	21	−5.9	8.4	17	0.8	8.8	0.02
DC visits	18	−3.1	8.3	16	1.8	8.6	0.10

*N*: number; SD: standard deviation; BMI: body mass index; SBP and DBP: systolic and diastolic blood pressure; PCP: primary care physician; DC: diabetes clinic.

**Table:** (a) Comparison between individual and group care in patients with type 1 diabetes

Parameter	Individual care			Group care			*p* value
	*N*	Mean	SD	*N*	Mean	SD	
HbA1c	11	−0.4	0.7	16	0.1	0.8	0.14
Weight	7	−2.4	4.2	14	0.1	1.2	0.15
BMI	7	−0.8	1.4	14	−0.1	1.2	0.15
SBP	8	5.5	15.2	14	9.2	15.4	0.59
DBP	8	1.5	9.7	14	5.2	8.4	0.36
Non-HDL C	11	−8.3	17	12	0.2	20.6	0.29
GP visits	9	−5.2	5.2	10	−1.2	8.1	0.22
DC visits	9	−3.6	6.0	9	−1.2	8.5	0.51
Hospital admission	11	−0.1	1.0	11	0.45	1.4	0.32
WHO-5	11	17.1	28.4	16	14.25	23.1	0.21
PAID	8	−0.2	0.2	15	0.1	0.4	0.03
PMD	8	0.2	0.4	15	0.03	0.6	0.71

**Table:** (b) Comparison between individual and group care in patients with type 2 diabetes

	Individual care			Group care			*p* value
	*N*	Mean	SD	*N*	Mean	SD	
HbA1c	13	0.10	0.70	16	−0.09	0.80	0.49
Weight	16	2.6	5.4	16	1.3	4.8	0.46
BMI	16	0.98	2.00	16	0.53	1.70	0.48
SBP	9	10.6	17.3	15	−1.5	12.5	0.15
DBP	9	−4.0	7.3	15	.2	8.7	0.24
Non-HDL C	10	−4.1	20.5	13	6.3	18.9	0.24
GP visits	12	−6.3	104.	7	3.7	9.5	0.052
DC visits	9	−2.6	10.5	7	5.66	7.4	0.10
Hospital admission	13	0.6	1.3	12	0.17	1.30	0.40
WHO-5	8	9.5	28.2	10	−3.6	14.3	0.54
PAID	8	0.16	0.60	11	0.02	0.57	0.59
PMD	7	−0.14	1.10	11	−0.14	0.70	0.98

*N*: number; SD: standard deviation; BMI: body mass index; SBP and DBP: systolic and diastolic blood pressure; Non-HDL C: non-HDL cholesterol; GP: general practitioner; DC: diabetes clinic; WHO-5: World Health Organization quality of life short version; PAID: patient areas in diabetes; PMD: personal models of diabetes questionnaires.
